# Supplementary material for: Evaluation of Consumer Perception of New Aquaculture Products through Applying Focus Group and Check-All-That-Apply Methodologies
Source: Foods. 2024 Aug 7;13(16):2480. doi: 10.3390/foods13162480 (PMC11353878; doi:10.3390/foods13162480)
Supplement: Supplementary file 1 [file foods-13-02480-s001.zip › foods-3099207-supplementary.pdf]

ANEXO 1

Survey Created Using RedJade Software

Questionnaire Page

CUESTIONARIO DE NUEVOS PRODUCTOS ACUÍCOLAS

Por favor, contesta las siguientes preguntas con la máxima sinceridad:

Questionnaire Page

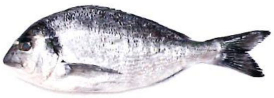

DORADA

1 Por favor, selecciona tu grado de aceptación sobre este producto

- ◯ Me disgusta muchísimo
- ◯ Me disgusta mucho
- ◯ Me disgusta bastante
- ◯ Me disgusta ligeramente
- ◯ Ni me gusta ni me disgusta
- ◯ Me gusta ligeramente
- ◯ Me gusta bastante
- ◯ Me gusta mucho
- ◯ Me gusta muchísimo

2 ¿Estarías dispuesto a comprar este producto?

Por favor, selecciona el valor que mejor describa tu intención de compra.

- ◯ Seguro que no
- ◯ Probablemente no
- ◯ Igual sí o igual no
- ◯ Probablemente sí
- ◯ Seguro que sí

3 De la siguiente lista de productos elaborados con DORADA, selecciona TODOS los que comprarías o consumirías:

- ☐ Filete fresco sin espinas, sin piel
- ☐ Lomo fresco sin espinas, sin piel
- ☐ Fresco abierto por la mitad sin espinas, sin piel
- ☐ Snack como aperitivo
- ☐ Topping seco salado (virutas para ensaladas y cremas)
- ☐ Brandada (crema untable de aceite y dorada)
- ☐ Conserva al natural
- ☐ Conservas elaboradas (con tomate, con aceite, etc)
- ☐ Paté
- ☐ Tacos marinados
- ☐ Listo para el microondas sin guarnición
- ☐ Listo para el microondas con guarnición
- ☐ Marinado y ahumado
- ☐ Encurtido en salmuera
- ☐ Guisos tradicionales en lata
- ☐ Guisos tradicionales para microondas
- ☐ Carpaccio (dorada cruda, cortada en filetes muy finos y aliñados)
- ☐ Embutidos curados

Questionnaire Page

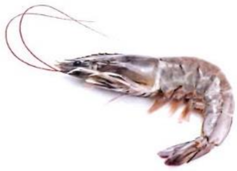

LANGOSTINO

4 Por favor, selecciona tu grado de aceptación sobre este producto (LANGOSTINO):

- ◯ Me disgusta muchísimo
- ◯ Me disgusta mucho
- ◯ Me disgusta bastante
- ◯ Me disgusta ligeramente
- ◯ Ni me gusta ni me disgusta
- ◯ Me gusta ligeramente
- ◯ Me gusta bastante
- ◯ Me gusta mucho
- ◯ Me gusta muchísimo

5 ¿Estarías dispuesto a comprar este producto (LANGOSTINO)?

Por favor, selecciona el valor que mejor describa tu intención de compra.

- ◯ Seguro que no
- ◯ Probablemente no
- ◯ Igual sí o igual no
- ◯ Probablemente sí
- ◯ Seguro que sí

6 De la siguiente lista de productos elaborados con LANGOSTINO, selecciona TODOS los que comprarías o consumirías:

- ☐ Filete fresco sin cáscara, sin cabeza
- ☐ Lomo fresco sin cáscara, sin cabeza
- ☐ Fresco abierto por la mitad sin cáscara, sin cabeza
- ☐ Snack como aperitivo
- ☐ Topping seco salado (tropezón para ensaladas y cremas)
- ☐ Brandada (crema untable de aceite y langostino)
- ☐ Conserva al natural
- ☐ Conservas elaboradas (con tomate, con aceite, etc)
- ☐ Paté
- ☐ Rodajas marinadas
- ☐ Listo para el microondas sin guarnición
- ☐ Listo para el microondas con guarnición
- ☐ Marinado y ahumado
- ☐ Encurtido en salmuera
- ☐ Guisos tradicionales en lata
- ☐ Guisos tradicionales para microondas
- ☐ Carpaccio (langostino crudo, cortado muy fino y aliñado)
- ☐ Embutidos curados

Questionnaire Page

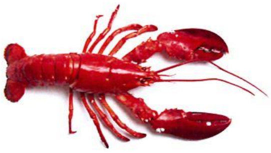

LANGOSTA

7 Por favor, selecciona tu grado de aceptación sobre este producto (LANGOSTA):

- Me disgusta muchísimo
- Me disgusta mucho
- Me disgusta bastante
- Me disgusta ligeramente
- Ni me gusta ni me disgusta
- Me gusta ligeramente
- Me gusta bastante
- Me gusta mucho
- Me gusta muchísimo

8 ¿Estarías dispuesto a comprar este producto (LANGOSTA)?

Por favor, selecciona el valor que mejor describa tu intención de compra.

- Seguro que no
- Probablemente no
- Igual sí o igual no
- Probablemente sí
- Seguro que sí

9 De la siguiente lista de productos elaborados con LANGOSTA, selecciona TODOS los que comprarías o consumirías:

- ☐ Filete fresco sin cáscara, sin cabeza
- ☐ Lomo fresco sin cáscara, sin cabeza
- ☐ Fresco abierto por la mitad sin cáscara, sin cabeza
- ☐ Snack como aperitivo
- ☐ Topping seco salado (virutas para ensaladas y cremas)
- ☐ Brandada (crema untable de aceite y langosta)
- ☐ Conserva al natural
- ☐ Conservas elaboradas (con tomate, con aceite, etc)
- ☐ Paté
- ☐ Rodajas marinadas
- ☐ Listo para el microondas sin guarnición
- ☐ Listo para el microondas con guarnición
- ☐ Marinado y ahumado
- ☐ Encurtido en salmuera
- ☐ Guisos tradicionales en lata
- ☐ Guisos tradicionales para microondas
- ☐ Carpaccio (langosta cruda, cortada muy fina y aliñada)
- ☐ Embutidos curados

Questionnaire Page

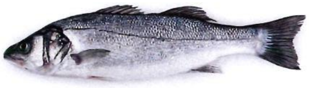

LUBINA

10 Por favor, selecciona tu grado de aceptación sobre este producto (LUBINA):

- ◯ Me disgusta muchísimo
- ◯ Me disgusta mucho
- ◯ Me disgusta bastante
- ◯ Me disgusta ligeramente
- ◯ Ni me gusta ni me disgusta
- ◯ Me gusta ligeramente
- ◯ Me gusta bastante
- ◯ Me gusta mucho
- ◯ Me gusta muchísimo

11 ¿Estarías dispuesto a comprar este producto (LUBINA)?

Por favor, selecciona el valor que mejor describa tu intención de compra.

- ◯ Seguro que no
- ◯ Probablemente no
- ◯ Igual sí o igual no
- ◯ Probablemente sí
- ◯ Seguro que sí

12 De la siguiente lista de productos elaborados con LUBINA, selecciona TODOS los que comprarías o consumirías:

- ☐ Filete fresco sin espinas, sin piel
- ☐ Lomo fresco sin espinas, sin piel
- ☐ Fresco abierto por la mitad sin espinas, sin piel
- ☐ Snack como aperitivo
- ☐ Topping seco salado (virutas para ensaladas y cremas)
- ☐ Brandada (crema untable de aceite y lubina)
- ☐ Conserva al natural
- ☐ Conservas elaboradas (con tomate, con aceite, etc)
- ☐ Paté
- ☐ Tacos marinados
- ☐ Listo para el microondas sin guarnición
- ☐ Listo para el microondas con guarnición
- ☐ Marinado y ahumado
- ☐ Encurtido en salmuera
- ☐ Guisos tradicionales en lata
- ☐ Guisos tradicionales para microondas
- ☐ Carpaccio (lubina cruda, cortada en filetes muy finos y aliñados)
- ☐ Embutidos curados

Questionnaire Page

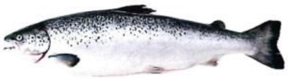

SALMÓN

13 Por favor, selecciona tu grado de aceptación respecto a este producto (SALMÓN):

- ◯ Me disgusta muchísimo
- ◯ Me disgusta mucho
- ◯ Me disgusta bastante
- ◯ Me disgusta ligeramente
- ◯ Ni me gusta ni me disgusta
- ◯ Me gusta ligeramente
- ◯ Me gusta bastante
- ◯ Me gusta mucho
- ◯ Me gusta muchísimo

14 ¿Estarías dispuesto a comprar este producto (SALMÓN)?

Por favor, selecciona el valor que mejor describa tu intención de compra.

- ◯ Seguro que no
- ◯ Probablemente no
- ◯ Igual sí o igual no
- ◯ Probablemente sí
- ◯ Seguro que sí

15 De la siguiente lista de productos elaborados con SALMÓN, selecciona TODOS los que comprarías o consumirías:

- [ ] Filete fresco sin espinas, sin piel
- [ ] Lomo fresco sin espinas, sin piel
- [ ] Fresco abierto por la mitad sin espinas, sin piel
- [ ] Snack como aperitivo
- [ ] Topping seco salado (virutas para ensaladas y cremas)
- [ ] Brandada (crema untable de aceite y salmon)
- [ ] Conserva al natural
- [ ] Conservas elaboradas (con tomate, con aceite, etc)
- [ ] Paté
- [ ] Tacos marinados
- [ ] Listo para el microondas sin guarnición
- [ ] Listo para el microondas con guarnición
- [ ] Marinado y ahumado
- [ ] Encurtido en salmuera
- [ ] Guisos tradicionales en lata
- [ ] Guisos tradicionales para microondas
- [ ] Carpaccio (salmón crudo, cortado en filetes muy finos y aliñados)
- [ ] Embutidos curados

Questionnaire Page

Casi hemos terminado. ¿Nos contestas unas preguntas?

16 **Por favor, especifica tu rango de edad.**

- ◯ 15 - 24
- ◯ 25 - 34
- ◯ 35 - 44
- ◯ 45 - 54
- ◯ 55 - 64
- ◯ 65 - 100

17 **Por favor, especifica tu género**

- ◯ Femenino
- ◯ Masculino
- ◯ No Binario

18 **¿Cuál es tu procedencia?**

- ◯ España
- ◯ Europa (excepto España)
- ◯ Latinoamérica
- ◯ Otros

19 **¿Cuál de las siguientes opciones se acerca más a tu nivel de estudios?**

- ◯ Educación básica obligatoria
- ◯ Bachillerato o FP
- ◯ Grado universitario
- ◯ Máster universitario/Doctorado

20 ¿Cuál de las siguientes opciones describe tu situación actual?

- Estudiante
- Desempleado/a
- Empleado/a a tiempo parcial
- Empleado/a a tiempo completo
- Jubilado

21 ¿Cuál de las siguientes opciones se corresponde con tu modo de vida?

- Vivo solo/a
- Vivo en pareja
- Vivo en familia
- Vivo en piso compartido (residencias de estudiantes, etc)
- Otros

22 ¿Cuál es tu presupuesto personal para comprar comida?

- Menos de 200 euros/mes
- 200 - 299 euros/mes
- 300- 399 euros/mes
- 400- 499 euros/mes
- Más de 500 euros/mes
- Prefiero no indicarlo

Questionnaire Page

Evaluación completada

Muchas gracias por tu participación.
